# Complete Genome Sequencing and Comparative Genomics of Three Potential Probiotic Strains, *Lacticaseibacillus casei* FBL6, *Lacticaseibacillus chiayiensis* FBL7, and *Lacticaseibacillus zeae* FBL8

**DOI:** 10.3389/fmicb.2021.794315

**Published:** 2022-01-07

**Authors:** Eiseul Kim, Seung-Min Yang, Dayoung Kim, Hae-Yeong Kim

**Affiliations:** Institute of Life Sciences & Resources and Department of Food Science and Biotechnology, Kyung Hee University, Yongin, South Korea

**Keywords:** *Lacticaseibacillus chiayiensis*, *Lacticaseibacillus zeae*, complete genome sequencing, comparative genomics, probiotic, *Lacticaseibacillus casei*

## Abstract

*Lacticaseibacillus casei*, *Lacticaseibacillus chiayiensis*, and *Lacticaseibacillus zeae* are very closely related *Lacticaseibacillus* species. *L. casei* has long been proposed as a probiotic, whereas studies on functional characterization for *L. chiayiensis* and *L. zeae* are some compared to *L*. *casei*. In this study, *L. casei* FBL6, *L. chiayiensis* FBL7, and *L. zeae* FBL8 were isolated from raw milk, and their probiotic properties were investigated. Genomic analysis demonstrated the role of *L. chiayiensis* and *L. zeae* as probiotic candidates. The three strains were tolerant to acid and bile salt, with inhibitory action against pathogenic bacterial strains and capacity of antioxidants. Complete genome sequences of the three strains were analyzed to highlight the probiotic properties at the genetic level, which results in the discovery of genes corresponding to phenotypic characterization. Moreover, genes known to confer probiotic characteristics were identified, including genes related to biosynthesis, defense machinery, adhesion, and stress adaptation. The comparative genomic analysis with other available genomes revealed 256, 214, and 32 unique genes for FBL6, FBL7, and FBL8, respectively. These genomes contained individual genes encoding proteins that are putatively involved in carbohydrate transport and metabolism, prokaryotic immune system for antiviral defense, and physiological control processes. In particular, *L*. *casei* FBL6 had a bacteriocin gene cluster that was not present in other genomes of *L*. *casei*, resulting in this strain may exhibit a wide range of antimicrobial activity compared to other *L*. *casei* strains. Our data can help us understand the probiotic functionalities of the three strains and suggest that *L. chiayiensis* and *L. zeae* species, which are closely related to *L. casei*, can also be considered as novel potential probiotic candidate strains.

## Introduction

Probiotics are defined as live microorganisms that benefit host health when ingested in adequate amounts (Ye et al., 2020). Lactic acid bacteria can be utilized as probiotics provided that they have the desired properties, such as safety assurance for consumers, stability, and persistence in the gastrointestinal tract (Ye et al., 2020). Moreover, the ability to inhibit pathogens’ growth, improve microbial balance in the intestines, and regulate mucosal and systemic immunity is required. Many strains belonging to lactic acid bacteria function as probiotics, and they also play a significant role in improving the quality of fermented foods (Zhang et al., 2018).

*Lacticaseibacillus casei*, *Lacticaseibacillus chiayiensis*, and *Lacticaseibacillus zeae* belong to the Lacticaseibacillus species and are very closely related phenotypically and genotypically along with *Lacticaseibacillus paracasei* and *Lacticaseibacillus rhamnosus* (Kim et al., 2020b). *L. casei* is one of the most studied lactic acid bacterial strains associated with probiotics and used in industrial and applied health applications (Hill et al., 2018). Strains considered to be probiotics have been proven to exhibit beneficial properties by promoting health associated with the regulation of immune response and improvement of atopic dermatitis (Mazé et al., 2010; Kang et al., 2017). *L. casei* is often used in the fermentation of dairy products, resulting in improved flavor and texture (Hill et al., 2018). *L. chiayiensis* was first isolated in 2018 from cow manure (Huang et al., 2018), and since then, this species has not been reported anymore. *L. zeae*, designated as an independent species from *L. casei* in 2020, has been said to exhibit antimicrobial activity against two strains of enterotoxigenic *Escherichia coli* (Zhou et al., 2014; Huang et al., 2020; Chen et al., 2021).

Whole-genome sequencing of potential probiotic strains ensures accurate taxonomic assignment while also providing genetic data concerning the presence of probiotic traits, possible metabolic activities, and safety assessment such as virulence factors, antibiotic resistance genes, and genetics related to hazardous metabolites (Guinane et al., 2016; Li et al., 2017). However, despite its importance for predicting the critical abilities of putative probiotic strains, the current genomic information of *L. zeae* and *L. chiayiensis* is fairly limited, resulting in impossible genetic access. Currently, one complete genome of *L. zeae* was reported but none for *L. chiayiensis*. However, the draft genome of *L*. *chiayiensis* has been reported in the literature (Huang et al., 2018). Also, reports on probiotic properties and genomic evidence to support their functionality are not yet available for the two species. Genomic analysis, together with experimental studies, is important for constructing a probiotic profile of a novel strain. Therefore, in this study, we investigated a phenotypic assay for *L. casei* FBL6, *L. chiayiensis* FBL7, and *L. zeae* FBL8 and identified related genes that are responsible for their probiotic properties. Moreover, to gain a better insight into the probiotic effects, the comparative genomic analysis was conducted among the newly sequenced three strains and publicly available genomes, as well as a comparison of genetic characteristics of the three species.

## Materials and Methods

### Isolation of Strains and Identification

Raw milk samples were randomly collected from eight different ranches in Gyeonggi-do, Korea. Prior to the collection of raw milk samples, the udder was washed with distilled water and dried. The samples were obtained and directly placed into sterile tubes and immediately transferred to the laboratory. For the isolation of lactic acid bacterial strains, the serially diluted milk samples were spread on an MRS agar plate (Difco, Becton & Dickinson, Sparks, MD, United States) and incubated at 37°C for 48 h under anaerobic conditions. The different individual colonies were randomly selected according to morphological differences and purified on MRS broth (Difco). All isolates were stored at −80°C in glycerol until probiotic characterization.

Genomic DNA of the presumptive *Lacticaseibacillus* strain was extracted using the DNeasy Blood & Tissue Kits (Qiagen, Hilden, Germany) following the manufacturer’s protocol to identify isolates. These strains were identified *via* 16S rRNA gene sequencing with 27F/1492R primer pairs. The identification of *L. casei*, *L. chiayiensis*, and *L. zeae* was performed using a real-time PCR method targeting unique genes for each species (Kim et al., 2020b,^2021^). Three isolates demonstrating higher probiotic activities compared with other isolates were finally selected and designated respectively as FBL6, FBL7, and FBL8.

### Genome Sequencing and Assembly

Cultured cells were harvested *via* centrifugation at 13,600 × *g* for 5 min. The total genomic DNA for genome sequencing was extracted using the Wizard Genomic DNA Purification Kit (Promega, Madison, WI, United States) following the manufacturer’s protocols. Genomic DNA was sheared into 10-kb fragments, and then SMRTbell libraries were prepared using the SMRTbell Template Prep Kit v. 1.0 (Pacific Bioscience, Menlo Park, CA, United States). Prepared libraries were sequenced on a PacBio Sequel I sequencer (Pacific Bioscience) using the Sequel Sequencing Kit v. 3.0 on single-molecule real-time sequencing technology cell 1M v. 2.0. The generated raw reads were filtered to remove adapter sequences and short reads. Filtered subreads were assembled through the hierarchical genome assembly process.

### Genomic Analysis

Genes of genomes were predicted using Prokka v. 1.12, and the corresponding function annotation was performed by Clusters of Orthologous Groups (COGs). The most important probiotic genes and stress-related genes were determined by the Rapid Annotations using Subsystem Technology and Prokka. Putative virulence factors were detected using VirulenceFinder v. 2.0, and antimicrobial resistance genes acquired within three genomes were identified using the Comprehensive Antibiotic Resistance Database (CARD) v. 3.1.1 and ResFinder v. 4.1. The pathogenicity of the three genomes was determined using PathogenFinder v. 1.1. Putative prophage insert regions were identified using the PHage Search Tool Enhanced Release (PHASTER) (Arndt et al., 2016). Clustered Regularly Interspaced Short Palindromic Repeats (CRISPR) and CRISPR-associated genes (Cas) were detected using CRISPRCasFinder (Couvin et al., 2018). Secondary metabolite biosynthesis gene clusters were identified using antiSMASH bacterial v. 5.0 (Blin et al., 2019). Carbohydrate-active enzymes (CAZymes) within three genomes were identified using the CAZy database.^1^

### Comparative Genomic Analysis

For the comparative genomic analysis, publicly available genome sequences of *L. casei*, *L. chiayiensis*, *L. zeae*, and other reported *Lacticaseibacillus* species, *L*. *paracasei*, and *L*. *rhamnosus*, were downloaded from the NCBI database (Table 1). The phylogenetic relatedness among the FBL6, FBL7, and FBL8 strains and other strains was investigated using 16S rRNA gene sequences, average nucleotide identity (ANI), and core and pan-genome sequences to infer their evolutionary relationships. 16S rRNA gene sequences were extracted from whole-genome sequences, and a phylogenetic tree was created using the neighbor-joining method. A phylogenetic identification by ANI analysis was conducted using orthologous average nucleotide identity (OrthoANI) (Lee et al., 2016). A phylogenetic tree based on pan-genome was constructed using the pan-genome workflow in the Anvi’o software v. 6.0 (Eren et al., 2015). Pan-genome and core-genome were analyzed using the Bacterial Pan-genome Analysis (BPGA) tool v. 1.3 with the default parameters. The assignment of COG functional categories for genes derived from core-genome and pan-genome was performed using USEARCH in the BPGA tool against the COG database.

**TABLE 1:** Genome features of *L. casei*, *L. chiayiensis*, and *L. zeae* used in this study.

Organism name	Strain	Size (Mb)	GC%	CDS	Accession no.	Level
*L. casei*	ATCC 393	2.95296	47.9	2585	AP012544.1	Complete
*L. casei*	LC5	3.13287	47.9	2813	CP017065.1	Complete
*L. casei*	MGB0470	2.94091	47.9	2566	CP064303.1	Complete
*L. casei*	FBL6	3.13829	47.9	2908	CP074377.1	Complete
*L. chiayiensis*	NCYUAS	2.87209	47.1	2648	MSSM01	Contig
*L. chiayiensis*	BCRC 18859	2.66164	47.3	2144	NOXN01	Contig
*L. chiayiensis*	FBL7	2.85541	47.2	2744	CP074378.1	Complete
*L. zeae*	KCTC 3804	3.11033	47.8	2787	BACQ01	Contig
*L. zeae*	DSM 20178	3.12134	47.7	2812	AZCT01	Scaffold
*L. zeae*	CRBIP24.58	3.09086	47.8	2751	VBWN01	Contig
*L. zeae*	CRBIP24.44	3.08327	47.7	2804	VBWO01	Contig
*L. zeae*	CECT 9104	3.07341	48.0	2753	LS991421.1	Complete
*L. zeae*	FBL8	3.13252	47.8	2967	CP074379.1	Complete
*L*. *paracasei*	ATCC 334	2.92433	46.6	2608	CP000423.1	Complete
*L*. *paracasei*	Zhang	2.89846	46.4	2631	CP001084.2	Complete
*L*. *paracasei*	BL23	3.0792	46.3	2884	FM177140.1	Complete
*L*. *paracasei*	8700:02:00	3.02535	46.3	2794	CP002391.1	Complete
*L*. *paracasei*	BD-II	3.12729	46.3	2922	CP002618.1	Complete
*L*. *paracasei*	LC2W	3.07743	46.4	2862	CP002616.1	Complete
*L*. *paracasei*	W56	3.1321	46.3	2843	HE970764.1	Complete
*L*. *paracasei*	LOCK919	3.14337	46.2	2928	CP005486.1	Complete
*L*. *paracasei*	N1115	3.06428	46.5	2798	CP007122.1	Complete
*L*. *paracasei*	JCM 8130	3.0178	46.6	2730	AP012541.1	Complete
*L*. *paracasei*	CAUH35	2.97335	46.3	2684	CP012187.1	Complete
*L*. *paracasei*	L9	3.07644	46.3	2807	CP012148.1	Complete
*L*. *paracasei*	KL1	2.91889	46.6	2620	CP013921.1	Complete
*L*. *paracasei*	IIA	3.24614	46.2	3001	CP014985.1	Complete
*L*. *paracasei*	TK1501	2.94254	46.5	2656	CP017716.1	Complete
*L*. *paracasei*	FAM18149	2.96971	46.3	2727	CP017261.1	Complete
*L*. *paracasei*	TMW 1.1434	3.17011	46.3	2803	CP016355.1	Complete
*L*. *paracasei*	EG9	3.07441	46.4	2811	CP029546.1	Complete
*L*. *paracasei*	Lpc10	3.05212	46.3	2801	CP029686.1	Complete
*L*. *paracasei*	ZFM54	3.04868	46.4	2802	CP032637.1	Complete
*L*. *paracasei*	AO356	3.09656	46.3	2956	CP025499.1	Complete
*L*. *paracasei*	IJH-SONE68	3.1812	46.4	2847	AP018392.1	Complete
*L*. *paracasei*	SRCM103299	3.18745	46.4	2922	CP035563.1	Complete
*L*. *paracasei*	CBA3611	3.10253	46.3	2872	CP041657.1	Complete
*L*. *paracasei*	NJ	3.08341	46.4	2772	CP041944.1	Complete
*L*. *paracasei*	10266	3.04491	46.4	2740	CP031785.1	Complete
*L*. *paracasei*	IBB3423	3.24058	46.3	2969	CP022954.1	Complete
*L*. *paracasei*	TD 062	2.86752	46.4	2555	CP044361.1	Complete
*L*. *paracasei*	CACC 566	3.2402	46.2	3015	CP048003.1	Complete
*L*. *paracasei*	347-16	3.21903	46.2	2990	CP052065.1	Complete
*L*. *paracasei*	Lp02	3.09226	46.3	2793	CP039707.1	Complete
*L*. *paracasei*	NFFJ04	3.19615	46.3	2979	CP050500.1	Complete
*L*. *paracasei*	TK-P4A	3.08027	46.4	2833	CP045567.1	Complete
*L*. *paracasei*	MGB0245	3.18613	46.3	2929	CP064299.1	Complete
*L*. *paracasei*	MGB0734	3.30028	46.2	3004	CP064304.1	Complete
*L*. *paracasei*	MGB0625	3.25115	46.3	2953	CP064311.1	Complete
*L*. *paracasei*	MGB0747	3.14566	46.3	2886	CP064314.1	Complete
*L*. *paracasei*	MGB0761	3.17238	46.2	2910	CP064316.1	Complete
*L*. *paracasei*	ZY-1	3.25423	46.4	2964	CP065154.1	Complete
*L*. *paracasei*	S-NA5	3.14156	46.4	2856	CP068408.1	Complete
*L*. *paracasei*	S-NB	3.15214	46.4	2868	CP068416.1	Complete
*L*. *paracasei*	HL182	3.14151	46.3	2854	CP072181.1	Complete
*L*. *paracasei*	2A	3.04223	46.3	2767	CP073916.1	Complete
*L*. *paracasei*	GR0548	3.01478	46.3	2753	CP078097.1	Complete
*L*. *rhamnosus*	ATCC 53103	3.00505	46.7	2687	AP011548.1	Complete
*L*. *rhamnosus*	GG (ATCC 53103)	3.01011	46.7	2703	FM179322.1	Complete
*L*. *rhamnosus*	Lc 705	3.03311	46.6	2652	FM179323.1	Complete
*L*. *rhamnosus*	ATCC 8530	2.96034	46.8	2623	CP003094.1	Complete
*L*. *rhamnosus*	LOCK900	2.88338	46.8	2586	CP005484.1	Complete
*L*. *rhamnosus*	LOCK908	2.9909	46.8	2666	CP005485.1	Complete
*L*. *rhamnosus*	LRB	2.93495	46.8	2428	CP016823.1	Complete
*L*. *rhamnosus*	BFE5264	3.11476	46.8	2785	CP014201.1	Complete
*L*. *rhamnosus*	Pen	2.88497	46.8	2570	CP020464.1	Complete
*L*. *rhamnosus*	4B15	3.04784	46.7	2727	CP021426.1	Complete
*L*. *rhamnosus*	LR5	2.97259	46.7	2655	CP017063.1	Complete
*L*. *rhamnosus*	DSM 14870	3.01315	46.7	2698	CP006804.1	Complete
*L*. *rhamnosus*	GG	3.01012	46.7	2699	CP031290.1	Complete
*L*. *rhamnosus*	LR-B1	3.0075	46.7	2677	CP025428.1	Complete
*L*. *rhamnosus*	hsryfm 1301	3.06781	46.8	2769	CP044228.1	Complete
*L*. *rhamnosus*	BIO6870	3.00671	46.7	2691	CP044506.1	Complete
*L*. *rhamnosus*	BIO5326	2.98957	46.8	2655	CP046267.1	Complete
*L*. *rhamnosus*	LV108	2.92366	46.8	2630	CP053619.1	Complete
*L*. *rhamnosus*	JL-1	3.0075	46.7	2686	CP046395.1	Complete
*L*. *rhamnosus*	TK-F8B	3.05844	46.7	2730	CP045586.1	Complete
*L*. *rhamnosus*	B6	2.92449	46.8	2647	CP067042.2	Complete
*L*. *rhamnosus*	LDTM7511	3.00747	46.7	2670	CP051227.1	Complete
*L*. *rhamnosus*	CE1	2.93517	46.8	2564	CP073317.1	Complete
*L*. *rhamnosus*	X253	2.99115	46.8	2653	CP073711.1	Complete
*L*. *rhamnosus*	AS	2.93512	46.8	2561	CP073915.1	Complete
*L*. *rhamnosus*	CAU 1365	2.99104	46.8	2658	CP082961.1	Complete
*L*. *rhamnosus*	PMC203	2.99312	46.7	2647	CP086326.1	Complete
*L*. *rhamnosus*	NCTC13710	2.99105	46.8	2661	LR134322.1	Complete
*L*. *rhamnosus*	NCTC13764	2.98839	46.8	2658	LR134331.1	Complete

### Evaluation of the Probiotic Properties of FBL6, FBL7, and FBL8

#### Acid and Bile Salt Tolerance

Tolerance to acid and bile salt was observed according to the previous studies (Yu et al., 2019; Jomehzadeh et al., 2020). Each strain was incubated in MRS broth at 37°C overnight. Aliquots (0.1 mL) of each active culture were inoculated in 10 mL MRS broth adjusted to pH 2.5 with 0.1 N hydrochloric acid (HCl) and then incubated for 3 h at 37°C. Each strain was inoculated in MRS broth containing 0.3% ox gall bile salt (Sigma-Aldrich, St. Louis, MO, United States) and incubated for 24 h at 37°C to confirm tolerance to bile salt. After incubation, cell suspension was spread on an MRS agar plate, and then viable cell count that survived at low pH and bile salt were enumerated by plate counting. *L. rhamnosus* American Type Culture Collection (ATCC) 53103 (LGG) was utilized as the control. The survival rate (%) of each strain was calculated using the following formula: (N/N_0_) × 100, where N and N_0_ denote viable cell numbers after culturing with low pH or bile salt and initial viable cell numbers, respectively.

#### Antimicrobial Activity

The antimicrobial activity of strains against pathogenic Gram-positive and Gram-negative bacterial strains was determined using the well-diffusion method (Jomehzadeh et al., 2020). *Listeria monocytogenes* ATCC 19111, *L. ivanovii* ATCC 19119, *Bacillus cereus* ATCC 21772, *E. coli* O157:H7 ATCC 43894, *E. coli* O1:K1:H7 ATCC 11775, and *Salmonella* Enteritidis ATCC 4931 were grown on nutrient broth (Difco) at 37°C overnight. The FBL6, FBL7, and FBL8 strains were grown in MRS broth at 37°C for 24 h and then centrifuged at 8,000 × *g* for 5 min to prepare a supernatant. The pathogens (10^6^ CFU/mL) were inoculated on Mueller–Hinton agar (Difco) and then cultured by putting the supernatant of the three strains in 8-mm-diameter wells. After incubation for 24 h at 37°C, antimicrobial activity was determined by measuring the inhibition zone surrounding the well.

#### Antioxidant Activity by Radical Scavenging Ability

The 2,2-diphenyl-1-picrylhydrazyl (DPPH) radical scavenging ability of the strains was analyzed according to the previous studies (Yu et al., 2019). The activated cell (200 μL) was mixed with 1 mL of 0.4 mM DPPH solution and reacted in a dark room for 30 min. After centrifugation at 8,000 × *g* for 5 min, the absorbance of each supernatant was measured at 517 nm. The control group used the same amount of distilled water as the sample, and 0.2 mM ascorbic acid was used as the positive control. Scavenging activity (%) was calculated using the following formula: [1 – (A_*sample*_/A_*control*_)] × 100, where A_*control*_ and A_*sample*_ indicate the absorbance of the control and absorbance of the sample, respectively.

#### Adhesion Ability

The adhesion ability was evaluated using the human intestinal epithelial adenocarcinoma cell line (HT-29) (Yu et al., 2019). The HT-29 cell was obtained from the ATCC (Manassas, VA, United States). The cells were cultured in Roswell Park Memorial Institute (RPMI) 1640 medium containing fetal bovine serum (FBS, 10%, v/v) at 37°C in a humidified atmosphere of 5% CO_2_ until a 70–80% confluent monolayer was obtained. The HT-29 cells (1 × 10^5^ cells/well) were seeded into a 24-well culture plate and cultured until a confluent monolayer was observed. After replacing the FBS-free RPMI 1640 medium, the isolates (1 × 10^8^ CFU/mL) were added to each well, with each strain being tested in triplicates and incubated for 2 h. Subsequently, each well was rinsed three times with PBS, and the attached cells were lysed with 0.1% Triton X-100 (Sigma-Aldrich). The number of adherent bacteria was counted by spreading the serial dilutions on MRS agar. The adhesin value (%) was calculated using the following formula: N_1_/N_0_ × 100, where N_0_ and N_1_ denote the initial viable cell numbers and viable cell numbers obtained from the HT-29 cells, respectively.

#### Autoaggregation and Coaggregation

Autoaggregation and coaggregation tests were conducted according to previous studies (Lee et al., 2014). Autoaggregation of strains was observed to indirectly confirm intestinal cell adhesion. The cell (4 mL) suspended in PBS (10^8^ CFU/mL) was mixed by vortexing for 20 s and then incubated at room temperature for 5 h to determine autoaggregation. Each time, 0.1 mL of each supernatant was mixed with 0.9 mL PBS, and absorbance was measured at 600 nm. The following formula was used to calculate autoaggregation of strain (%): A0–(At/A0) × 100, where At and A0 denote the absorbance at 5 h and absorbance at 0 h, respectively.

For the measurement of coaggregation, the cell suspensions of each strain and pathogenic strain were mixed and then vortexed for 20 s. The control tubes were prepared using 4 mL of putative probiotic strain and pathogenic strain in each tube. After incubation for 5 h, the absorbance of the suspension was measured at 600 nm. The following formula was used to calculate the coaggregation of a strain: {[(A_*x*_ + A_*y*_)/2] – A_*mix*_/(A_*x*_ + A_*y*_)/2} × 100, where A_*x*_ and A_*y*_ denote the bacterial suspension in the control tube, and A_*mix*_ indicates the mixed bacterial suspension at 5 h.

#### Statistical Analysis

All experiments for probiotic properties were conducted in triplicate, and the statistical analysis of values was performed using R version 4.1.0. Significant differences (*p* < 0.05) between the mean ± standard deviation values were determined by one-way analysis of variance and Duncan’s multiple range test.

## Results and Discussion

### General Genome Features

The complete genome of *L. casei* FBL6 contained a chromosome of 3,138,294 bp with a guanine-cytosine (GC) content of 47.9% (Supplementary Table 1). The complete genomes of *L. chiayiensis* FBL7 and *L. zeae* FBL8 contained a chromosome of 2,855,405 bp with a GC content of 47.2% and a chromosome of 3,132,522 bp with a GC content of 47.8%, respectively. Previous studies have reported that strains occurring in host-associated environments, such as the gut and oral, showed small genome size and gene number, whereas strains present in soil have the largest genome size (Bentkowski et al., 2015; Cobo-Simón and Tamames, 2017; Jia et al., 2022). They also reported a positive correlation between genome size and ubiquity, suggesting that microorganisms with larger genome size can adapt to more environmental conditions. FBL6, FBL7, and FBL8 had a larger genome size and higher GC content than *L. acidophilus* NCFM (1,993,564 bp, 34.7%), *L. helveticus* CAUH18 (2,160,583 bp, 36.8%), and *L. johnsonii* ZLJ010 (1,999,879 bp, 34.91%) but were similar to *L. plantarum* EM (3,649,371 bp, 44.14%), *L. paracasei* ZFM54 (3,048,677 bp, 46.35%), and *L. rhamnosus* GG (3,010,111 bp, 46.7%) (Altermann et al., 2005; Kankainen et al., 2009; Yang et al., 2016; Zhang et al., 2019; Kim et al., 2020a; Qureshi et al., 2020). Among these, *L. plantarum*, which is ecologically flexible, has a large genome size (Kim et al., 2020a), similar to *L. casei*, *L. chiayiensis*, and *L. zeae*, indicating that the latter can adapt well to various environments.

In each genome, 2,908 coding genes of FBL6, 2,744 genes of FBL7, and 2,967 genes of FBL8 were assigned to COG families comprising 20 functional categories. In the three genomes, most coding genes were classified for general function prediction only (R, 13.11–13.43%), carbohydrate transport and metabolism (G, 11.68–13.01%), transcription (K, 8.87–9.19%), and amino acid transport and metabolism (E, 8.78–8.97%) (Supplementary Figure 1). The high proportion of genes involved in carbohydrate and amino acid metabolism indicates that three strains might utilize and degrade a wide range of carbohydrates and proteins (Heo et al., 2021).

### Phylogenetic Comparison of *L. casei*, *L. chiayiensis*, and *L. zeae*

**FIGURE 1:**
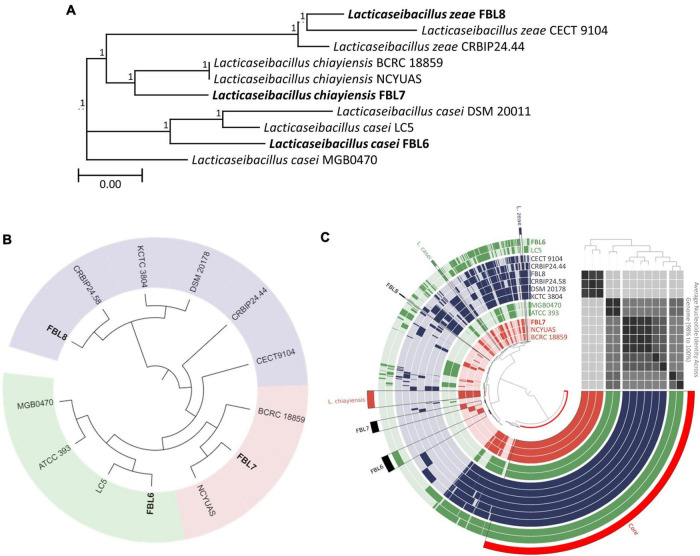
Phylogeny analysis based on **(A)** 16S rRNA gene sequences and **(B)** core-genome and **(C)** pan-genome. In tree based on pan-genome distribution, each ring represents one genome. The green, blue, and red rings indicate the *L. casei*, *L. chiayiensis*, and *L. zeae*, respectively. The tinted bright and dark color in ring represent gene absence and presence, respectively. ANI values were plotted as heatmap determined at high similarity (black) and low similarity (gray).

Phylogenetic comparison based on the 16S rRNA gene sequences demonstrated that *L. casei*, *L. chiayiensis*, and *L. zeae* were clustered according to the species (Figure 1A). In a phylogenetic tree based on the 16S rRNA gene sequences, FBL6 demonstrated a 99.62% similarity to other *L. casei* strains. In addition, it showed a high degree of similarity to *L. chiayiensis* (99.43%) and *L. zeae* (99.36%). FBL7 exhibited a 99.81% similarity to other *L. chiayiensis* strains and a high degree of similarity to *L. zeae* (99.49%). In some *L. zeae* strains, the 16S rRNA gene was present as a short fragment; thus, the remaining strains except these were compared with the 16S rRNA gene of FBL8. As a result, FBL8 demonstrated a high similarity (99.87%) to the *L. zeae* CECT9104 and *L. zeae* CRBIP24.44 strains.

According to the orthoANI calculation, FBL6 shared 95.36–95.81% sequence similarity to other *L. casei* genomes and 88.09–93.75% to *L. chiayiensis* and *L. zeae* genomes (Supplementary Table 2). *L. casei* FBL6 demonstrated a closer relationship with LC5. *L. casei* LC5 is a probiotic strain isolated from fermented dairy products and used as commercial probiotics (Kang et al., 2017). This strain has a probiotic effect on atopic dermatitis. FBL7 demonstrated 99.24 and 98.67% similarity to the genomes of *L. chiayiensis* NCYUAS and BCRC 18859, respectively. FBL8 shared 96.65–99.56% sequence similarities to other *L. zeae* genomes and high sequence similarities to other *L. casei* and *L. chiayiensis* genomes (88.12–94.38%). Also, in the phylogenetic analysis based on core-genome and pan-genome, FBL6, FBL7, and FBL8 were classified as *L. casei*, *L. chiayiensis*, and *L. zeae* and demonstrated a closer relationship with *L. casei* LC5, *L. chiayiensis* CRBLP24.58, and *L. zeae* NCYUAS, respectively (Figures 1B,C). The three species showed a high similarity to the 16S rRNA gene, which is more than 99.17% of each other, and it was confirmed that this gene could not be distinguished. Conversely, the analysis based on the whole-genome sequence clearly distinguishes the three species, suggesting that the genome sequence can help in achieving better understanding of the evolutionary history than the 16S rRNA gene.

### Pan-Genome and Core-Genome

**FIGURE 2:**
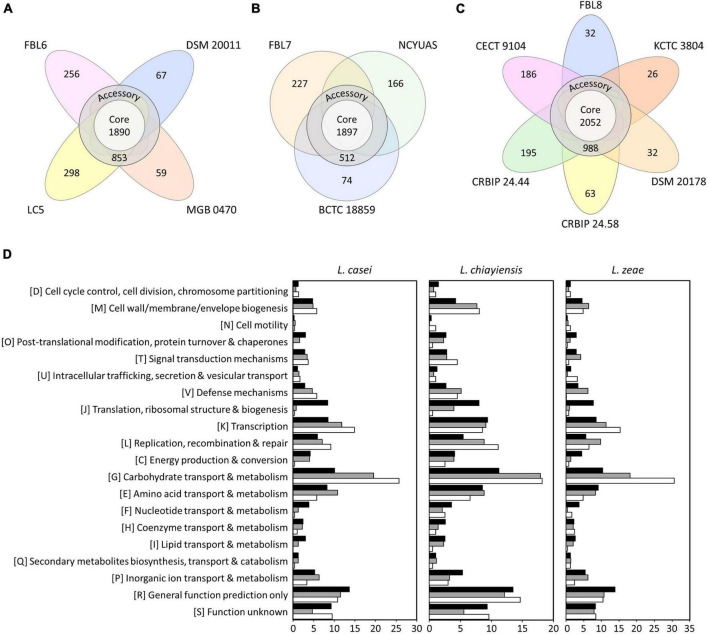
The number of core, accessory, and unique genes among **(A)** *L. casei*, **(B)** *L. chiayiensis*, and **(C)** *L. zeae* genomes, and **(D)** the number of genes assigned in cluster of orthologous group (COG) categories. Black, gray, and white bars represent COGs of the core, accessory, and unique genes, respectively.

Pan-genome and core-genome are used for investigating genomic diversity within closely related microorganisms or the same species (Zhang et al., 2019). Pan-genome is the entire set of genes of all strains that reflects the receptive capacity of the genetic determinants (Zhang et al., 2019). A core-genome is a set of genes shared by all strains in a species, including genetic determinants, for maintaining the characteristics of species (Bosi et al., 2016; Zhang et al., 2019). Four genomes of *L. casei* had a pan-genome of 3,423 orthologs, which were divided into a core-genome of 1,890 orthologs (55.21%), an accessory-genome of 853 orthologs (24.92%), and a unique genome of 680 orthologs (19.87%) (Figure 2). The pan-genome size was 1.24 times the average size of these four genomes, and the core-genome constituted approximately 68.84% of each *L. casei* genome. In the core-genome, 1,570 orthologs could be assigned in 20 COG categories. Some highly conserved functional categories, such as general function prediction only (R, 13.65%), carbohydrate transport and metabolism (G, 10.06%), and function unknown (S, 9.21%), were enriched among the core-genome of *L. casei* (Figure 2).

Three genomes of *L. chiayiensis* had a pan-genome of 2,876 orthologs, divided into a core-genome of 1,897 orthologs (65.96%), an accessory-genome of 512 orthologs (17.80%), and a unique genome of 467 orthologs (16.24%) (Figure 2). In the *L. chiayiensis* core-genome, 1,523 ortholog gene clusters could be assigned in 20 COG categories. Some highly conserved functional categories, such as general function prediction only (R, 13.48%), carbohydrate transport and metabolism (G, 11.20%), and transcription (K, 9.39%), were enriched among the core-genome of *L. zeae*.

Six genomes of *L. zeae* had a pan-genome of 3,574 orthologs, which were divided into a core-genome of 2,052 orthologs (57.41%), an accessory-genome of 988 orthologs (27.64%), and a unique genome of 534 orthologs (14.94%) (Figure 2). In the core-genome, 1,687 orthologs could be assigned in 20 COG categories. Some highly conserved functional categories, such as general function prediction only (R, 13.84%), carbohydrate transport and metabolism (G, 10.32%), and amino acid transport and metabolism (E, 9.12%), were enriched among the core-genome of *L. zeae*. Other *Lactobacillus* species, such as *L. johnsonii* and *L. ruminis*, which have a relatively small genome size, had a high proportion of genes related to core functions, such as translation, replication, and cell cycle control (Kant et al., 2017; Zhang et al., 2019). Contrarily, owing to its large genome size, *L. casei*, *L. chiayiensis*, and *L. zeae* had a relatively high number of proteins for amino acids and carbohydrate metabolisms, similar to *L. plantarum* (Boekhorst et al., 2004; Zhang et al., 2019).

### Unique Genome Characteristics

#### Unique Genome of *L. casei* FBL6

*Lacticaseibacillus casei* FBL6 had 256 unique genes, including 126 proteins with known functions and 130 hypothetical proteins of unknown function (Supplementary Table 3). To further investigate the function of the encoded protein of unique genes, they were assigned to the COG categories. Of the 256 individual genes in the FBL6 genomes, 100 were assigned to 13 COG categories (Supplementary Figure 2). In total, 24 genes were enriched in carbohydrate transport and metabolism (G, 24%). It contains genes such as PTS sugar transporter subunits IIB and IIA and PTS lactose/cellobiose transporter subunit IIA. These individual genes may endow FBL6 with probiotic strain properties that utilize various carbohydrates (Zhang et al., 2019). A total of 16 genes in the FBL6 genome were assigned to replication, recombination, and repair (L, 16%), containing genes associated with mobile elements. Furthermore, 16 genes were assigned in transcription (K, 16%), containing several transcriptional regulators. Previous study has reported that the presence of these transcriptional regulators can act on specific genes to regulate the expression and give them several benefits when they are present in an environment such as the gut (Zhang et al., 2019). However, further research is required to determine their association with genes and probiotics affected by transcriptional regulators.

#### Unique Genome of *L. chiayiensis* FBL7

*Lacticaseibacillus chiayiensis* FBL7 had 214 unique genes, including 140 proteins with known functions and 87 hypothetical proteins (Supplementary Table 4). Of the 214 unique genes, 73 were assigned to 17 COG categories (Supplementary Figure 2). A total of 13 genes were abundant in replication, recombination, and repair (L, 17.8%). In FBL7, CRISPR-associated protein Cas4 (locus_00047), which is not found in other *L. chiayiensis* strains, was present. This gene is one of the core CRISPR-associated (Cas) proteins involved in the prokaryotic immune system for antiviral defense (Zhang et al., 2012). Owing to the presence of this protein, FBL7 may be protected against mobile elements, including viruses, but other *L. chiayiensis* strains may not. Nine genes were assigned to carbohydrate transport and metabolism (G, 12.34%) and nine also to amino acid transport and metabolism (E, 12.34%). These unique genes endow FBL7 with the ability to obtain extra resources and protect itself against adverse stimuli, allowing better adaptation to various environments (Zhang et al., 2019).

#### Unique Genome of *L. zeae* FBL8

*Lacticaseibacillus zeae* FBL8 had 32 unique genes, including 26 proteins with known functions and 6 hypothetical proteins (Supplementary Table 5). Of the 32 unique genes, 17 were assigned to 11 COG functional categories (Supplementary Figure 2). Three genes were assigned to replication, recombination, and repair (L, 17.65%), containing several transposases and mobile element proteins. Two genes were assigned to the cell wall, membrane, and envelopment biogenesis (M, 11.76%). Two genes were assigned to signal transduction mechanisms (T, 11.76%), including two-component system sensors, histidine kinase, and response regulator. For lactic acid bacteria to survive while passing through the gastrointestinal tract, they need to have the ability to sense and respond to conditions in various changing environments (Zhang et al., 2019). They are generally detected by a two-component system (Cui and Qu, 2011), which consists of a membrane-associated histidine kinase that detects specific environmental signals and a cytoplasmic response regulator regulating gene expression (Cui and Qu, 2011). FBL8 carried a distinguished two-component system sensor histidine kinase. Thus, this unique gene might confer FBL8 to control various physiological processes, such as stress and toxicity.

### Comparative Genomic Analysis of *Lacticaseibacillus* Strains

Comparative genomic analysis was performed to compare the genomic characteristics between these three strains and other reported 83 *Lacticaseibacillus* strains. Of the 237,017 coding genes, 896 genes were shared by all genomes, comprising the core-genome. Accessory-genome is composed of 5,467 coding genes, and 1,642 coding genes were found only in a single genome. The pan- and core-genome were annotated COG database and then assigned to functional categories. Some highly conserved functional categories, such as translation, ribosomal structure and biogenesis (J, 12.71%), general function prediction only (R, 12.35%), function unknown (S, 10.45%), and amino acid transport and metabolism (E, 7.4%), were enriched among the core-genome of *Lacticaseibacillus* strains (Supplementary Figure 3). Translation functions are important for maintaining physiological parameters during changes to which gut microorganisms are exposed, suggesting their adaptive and survival roles (Sharma et al., 2018). Similar to our study, genomic analysis of *Bifidobacterium* showed high conservation of “Translation, ribosomal structure, and biogenesis (J)” class in the core-genome, demonstrating the importance of information storage for these bacteria to adapt and survive in the gut (Sharma et al., 2018). In addition, for the accessory-genome and unique-genome, we observed enrichment of genes in the COG category “Carbohydrate transport and metabolism (G, 17.73–17.52%).” Among the 1,642 unique genes, 65, 70, and 8 coding genes were specific to *L*. *casei* FBL6, *L*. *chiayiensis* FBL7, and *L*. *zeae* FBL8, respectively. These unique genes include MucBP domain-containing protein, LysM peptidoglycan-binding domain-containing protein, and hypothetical proteins.

### Probiotic-Related Genes

#### Lifestyle Adaptation to Stress

Lactic acid bacteria, which are widely used in food fermentation, need to survive in various environmental conditions, including pH, salinity, and temperature (Ye et al., 2020). Resistance to environmental conditions is one of the desirable properties of lactic acid bacteria. Proteins of adaptation to stress regulate evolution tolerance and demonstrate genetic adaptation (Ye et al., 2020).

*L. casei* FBL6, *L. chiayiensis* FBL7, and *L. zeae* FBL8 contained several genes encoding stress-related proteins (Table 2). The FBL6 genome harbored the eight genes (locus_00683 to locus_00690), which encodes F0F1 ATP synthetase, and the gene locus_01410, which encodes the cholylglycine hydrolase; these proteins are associated with resistance to low pH and bile. Cholylglycine hydrolase is responsible for converting conjugated bile acid into free bile acid and confers probiotics in the gastrointestinal tract (Heo et al., 2021). FBL6 genome possessed genes encoding heat stress proteins, including molecular chaperone (locus_02010), dnaK (locus_00321), GroES (locus_02686), GroEL (locus_02687), and heat shock protein GrpE (locus_00320). Furthermore, this genome has an ABC transport system (locus_01791 to locus_01794) and glycine betaine ABC transport system (locus_02770 to locus_02772), which may have an osmotic pressure function (Heo et al., 2021). FBL6 may be involved in oxidative stress resistance by encoding proteins such as thiol peroxidase (locus_01096), thioredoxin reductase (locus_00895), and glutaredoxin (locus_00073), which catalyze glutathione-dependent disulfide reductions (Zhang et al., 2019). FBL6, FBL7, and FBL8 contained genes encoding stress-related proteins, such as bile, acid, and oxidative stress tolerance genes. Moreover, two genomes carried genes that encode proteases related to stress response, heat stress proteins. These results suggest that three strains might resist multiple stress conditions and thus reach the intestines through the gastrointestinal tract.

**TABLE 2:** Genes coding for proteins involved in stress resistance.

Stress response	Product	FBL6	FBL7	FBL8
Bile resistance	Choloylglycine hydrolase family protein	locus_01410	locus_02697	locus_00436
Acid resistance	ATP synthase F0 sector subunit a	locus_00690	locus_00547	locus_01156
	ATP synthase F0 sector subunit c	locus_00689	locus_00548	locus_01157
	ATP synthase F0 sector subunit b	locus_00688	locus_00549	locus_01158
	ATP synthase subunit delta	locus_00687	locus_00550	locus_01159
	ATP synthase subunit alpha	locus_00686	locus_00551	locus_01160
	ATP synthase gamma chain	locus_00685	locus_00552	locus_01161
	ATP synthase subunit beta	locus_00684	locus_00553	locus_01162
	ATP synthase epsilon chain	locus_00683	locus_00554	locus_01163
Heat stress	Molecular chaperone	locus_02010	locus_00102	locus_00670
	Chaperone protein DnaK	locus_00321	locus_00937	locus_01529
	Heat shock protein 10 kDa family chaperone GroES	locus_02686	locus_01554	locus_02145
	Heat shock protein 60 kDa family chaperone GroEL	locus_02687	locus_01553	locus_02144
	Heat shock protein GrpE	locus_00320	locus_00938	locus_01530
Oxidative stress	Thiol peroxidase, Tpx-type	locus_01096	locus_00148	locus_00723
	Thioredoxin reductase	locus_00895	locus_00225	locus_00806
	Glutaredoxin related protein	locus_00073	locus_01187	locus_01788
Osmotic pressure	Choline ABC transport system, ATP-binding protein OpuBA	locus_01791	locus_02395	locus_00076
	Choline ABC transport system, permease protein OpuBB	locus_01792	locus_02394	locus_00075
	Choline ABC transport system, choline-binding protein OpuBC	locus_01793	locus_02393	locus_00074
	Choline ABC transport system, permease protein OpuBD	locus_01794	locus_02392	locus_00073
	Glycine betaine ABC transport system, ATP-binding protein OpuAA	locus_02770	locus_01474	locus_02058
	Glycine betaine ABC transport system, permease protein OpuAB	locus_02771	locus_01473	locus_02057
	Glycine betaine ABC transport system, glycine betaine-binding protein OpuAC	locus_02772	locus_01472	locus_02056
Adhesion	Translation elongation factor Tu	locus_00531	locus_00720	locus_01319
	Sortase, LPXTG specific	locus_02776	locus_01468	locus_02052
	Tyrosine-protein kinase transmembrane modulator EpsC	locus_02868	locus_01384	locus_01909
	Tyrosine-protein kinase EpsD	locus_02869	locus_01383	locus_01965
	Tyrosine-protein kinase transmembrane modulator EpsC	locus_02925	locus_01310	locus_01966
	Putative glycosyltransferase EpsH	locus_00767	locus_00473	locus_00265
	putative glycosyltransferase EpsJ	locus_01171	locus_00072	locus_00640
	Putative glycosyltransferase EpsH	locus_01607	locus_01313	locus_01082
	putative sugar transferase EpsL	locus_02878	locus_01369	locus_01959
	Putative glycosyltransferase EpsH	locus_02922	locus_02552	locus_01912
	Transmembrane protein EpsG	locus_02927	locus_01308	locus_01907

Regulatory proteins such as transcriptional regulators and sigma factors are important for microorganisms to adapt to different environments (Zhang et al., 2019). FBL6 had genes encoding several proteins involved in transcriptional regulation, including 110 transcriptional regulators and one sigma factor (RpoD). FBL7 and FBL8 had 100 and 105 transcriptional regulators, respectively, and one sigma factor in both strains. These genomes had fewer regulatory proteins than other probiotic species, *L. johnsonii*, and similar to *L. plantarum* (Zhang et al., 2018, 2019). A previous study has demonstrated that many regulatory proteins are contained in large genomes, contributing to various lifestyle differences (Zhang et al., 2019). Thus, *L. johnsonii* does not require a complex regulatory system, whereas *L. casei*, *L. chiayiensis*, and *L. zeae*, with their free-living lifestyle, appear to have regulatory machinery that can adapt to various environmental niches (Zhang et al., 2019).

#### Adhesion

Adhesion to the intestinal mucosa epithelium is a prerequisite for demonstrating a probiotic effect, and bacterial surface proteins are known to be involved in the colonization of the intestinal mucosa (Ye et al., 2020). The potential surface exposure protein of lactic acid bacteria plays a significant role in bacterial interactions with the environment or host (Ye et al., 2020). *L. casei* FBL6 encodes cell-surface proteins, such as lipoprotein signal peptide (locus_00412) and elongation factor Tu (locus_00531) (Table 2). In addition, in this genome, coding genes, such as LPXTG-specific sortase (locus_00278) and exopolysaccharides (*eps*) cluster, which are related to strain adhesion to the surrounding epithelial tissue, were contained within the FBL6 genome. Exopolysaccharide contributes strains and host interactions within the intestinal epithelial cells and mucosa and is involved in certain essential functions of probiotics, such as immunomodulation, antioxidation, aggregation, and biofilm formation (Alayande et al., 2020). Sortase-dependent proteins are cell-surface proteins in lactic acid bacteria and play a significant role in adhesion (Alayande et al., 2020). Therefore, the adhesion-related genes identified in the FBL6 genome may provide stability of strain and prolong its antagonistic effect on unwanted gut microorganisms, aiding in the effective colonization of the intestinal environment and inhibition of pathogens. Similarly, genes related to adhesion were also present in *L. chiayiensis* FBL7 and *L. zeae* FBL8.

#### Safety Assessment

One of the essential requirements for probiotics is their safety. Probiotics should be least resistant to critical antibiotics, as antibiotic resistance in bacteria can be inherent or acquired through mutation or horizontal gene transfer (Soni et al., 2021). The search using ResFinder and CARD returned no hits for antibiotic resistance genes in *L. casei* FBL6, *L. chiayiensis* FBL7, and *L. zeae* FBL8 genomes. As a result of the strain’s pathogenicity investigation, the calculated matched pathogenic families for FBL6 were 0, and the unmatched pathogenic families were 83. The probability that the strain is a human pathogen is 0.218; thus, this strain was predicted not to be a pathogen. These results suggest that this depicts the safety of the strain and reduces the risk of transferring antibiotic resistance genes to the host’s native gut microbiota. Also, FBL7 and FBL8 did not contain pathogenicity factors. The probability of being a pathogen in the three genomes was very low (FBL7, 0.164; FBL8, 0.213), and these strains were predicted to be non-human pathogens. Therefore, FBL6, FBL7, and FBL8 strains are safe.

### Mobile Genetic Element Analysis

PHAST predicted one intact prophage element and questionable prophage element in *L. casei* FBL6 and *L. chiayiensis* FBL7, respectively, and two intact prophage elements in *L. zeae* FBL8. Prophages are often detected in the genomes of probiotic *Lactobacillus* species (Stefanovic and McAuliffe, 2018; Feyereisen et al., 2019; Zhang et al., 2019). The prophage region in FBL6 extended from 1,379,286 to 1,410,750 bp and contained 46 proteins with an intact prophage element from locus_01305 to locus_01350 (Supplementary Table 6); this was the part of the prophage BH1 detected in *Lactobacillus* (accession no. NC_048737). For FBL7, the prophage region contained 52 proteins with a questionable prophage element (Supplementary Table 7). The genes in the prophage region were homologous to the genes of bacteriophage T25 (NC_048625) observed in *Lactobacillus*. For FBL8, the prophage 1 region contained 50 proteins with an intact prophage element, and prophage 2 contained 19 proteins with an intact prophage element (Supplementary Table 8). The genes in prophage 1 and 2 regions were homologous to a gene of phage BH1 (NC_048737) found in *Lactobacillus* and a gene of phage StauST398 (NC_023499) found in *Staphylococcus*, respectively. In these bacteriophage regions, virulence factors or genes related to pathogenic properties could not be detected.

CRISPR refers to a family of DNA repeats providing acquired immunity to foreign genetic elements (Zhang et al., 2019). These are composed of short and highly conserved repeats, interspaced by spacers, which are variable sequences, and often exist adjacent to the *cas* genes. The presence of these CRISPR loci increases the genomic stability of strains, allowing them to adapt to various environments. The FBL6 genome contained two CRISPR loci (Supplementary Table 9). The genomes of FBL7 and FBL8 harbored CRISPR regions, with associated spacer and Cas-gene. The FBL7 genome contained three CRISPR loci. The detected CRISPR Cas system was of type IIA (*cas1*, *cas9*, and *csn2*) and type IC (*cas1*, *cas2*, *cas3*, *cas4*, *cas5*, *cas7*, and *cas8*). The FBL8 genome contained five CRISPR loci. The detected CRISPR/CRISPR-associated (Cas) system was of type IC (*cas1*, *cas2*, *cas3*, *cas4*, *cas5*, *cas7*, and *cas8*). The presence of these CRISPR loci will provide the strains with the ability to defend themselves against plasmids, phages, and insertion sequences (Alayande et al., 2020). Therefore, the CRISPR Cas system could prevent *L. casei*, *L. chiayiensis*, and *L. zeae* from acquiring virulence or antibiotic resistance genes through horizontal gene transfer.

### Secondary Metabolic Product

**FIGURE 3:**
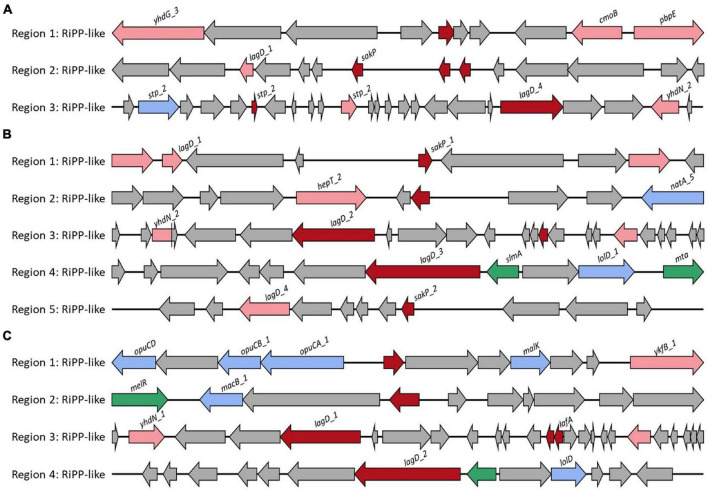
Biosynthetic gene cluster of secondary metabolites. Gene clusters for the biosynthesis of bacteriocin in **(A)** *L. casei* FBL6, **(B)** *L. chiayiensis* FBL7, and **(C)** *L. zeae* FBL8. The red, pink, blue, green, and gray arrows represent core biosynthetic genes, additional biosynthetic genes, transport-related genes, regulatory genes, and other genes, respectively.

The probiotic strain should survive in the intestine by competing with other microorganisms after adhesion or colonization in the gut (Heo et al., 2021). Strains with the ability to produce bacteriocin have the advantage of competing with other gut microorganisms (Heo et al., 2021). *L. casei* FBL6 encoded three secondary metabolic enzymes, ribosomally synthesized, and post-translationally modified peptide product (RiPP) cluster, all of which were predicted to produce bacteriocin. The first RiPP-like cluster demonstrated high similarity to bacteriocin IIc (Figure 3A). Some core biosynthetic genes and other genes in this cluster were specifically present only in FBL6 but not in different *L. casei* strains (Supplementary Table 10). These genes were helix–turn–helix transcriptional regulators, Blp family class II bacteriocin. In the second RiPP cluster, a gene encoding bacteriocin sakacin-P was only present in FBL6. Sakacins, class II bacteriocin, were first identified in *L. sakei* and demonstrated a bactericidal effect on food pathogens and spoilage species (Fallico et al., 2011). In particular, it has been reported that sakacin-P has relatively high activity against *L. monocytogenes* compared with other bacteriocins and is therefore promising for use in the food industry (Møretrø et al., 2000). Due to the presence of this gene, FBL6 may have antimicrobial activity against pathogens, but other *L. casei* strains may not. Moreover, the Rrf2 family transcriptional regulator, ABC transporter permease, and transporter-related genes in the third RiPP cluster were specifically present only in the FBL6 genome. *L. chiayiensis* FBL7 encoded five RiPP clusters, all of which were predicted to be bacteriocins, such as sakacin-P and bacteriocin IIc (Figure 3B). Unlike other *L. chiayiensis* strains, some transcriptional regulators, DNA-binding proteins, and hypothetical proteins were only present in the FBL7 genome (Supplementary Table 11). *L. zeae* FBL8 encoded four RiPP clusters, all of which were predicted to be bacteriocins (Figure 3C). The RiPP clusters in FBL8 were also present in other *L. zeae* genomes (Supplementary Table 12).

### Carbohydrate-Active Enzymes

**FIGURE 4:**
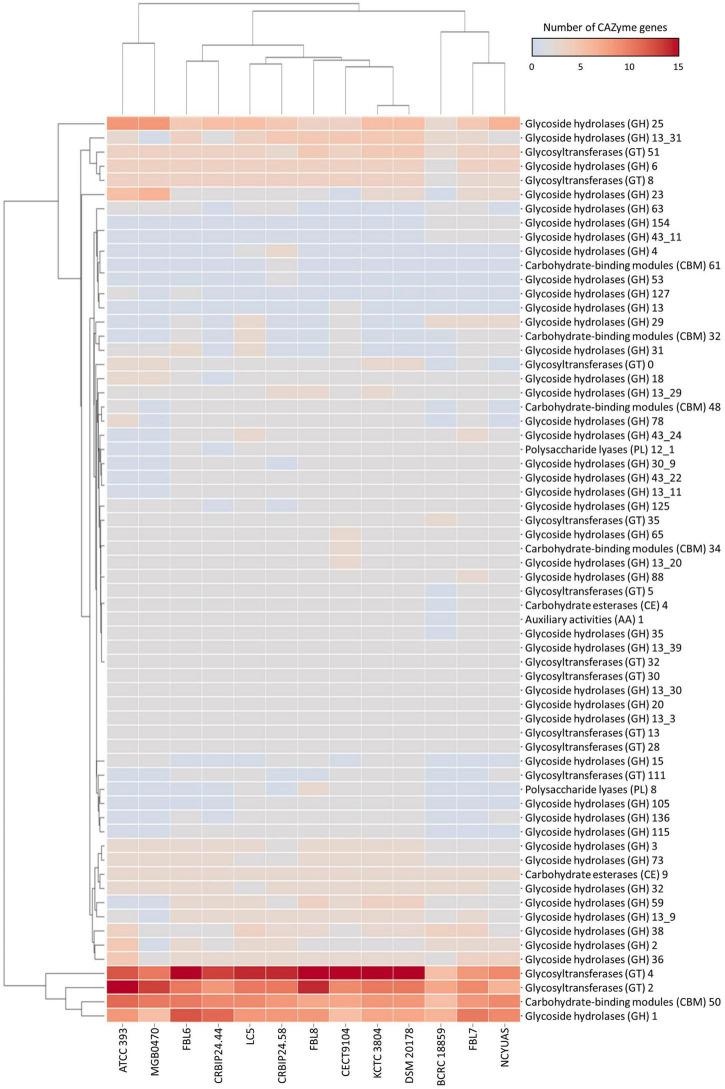
Heatmap for distribution of the number of genes of carbohydrate-active enzymes in *L. casei* FBL6, *L. chiayiensis* FBL7, *L. zeae* FBL8, and other strains. The right and bottom of the heatmap present carbohydrate-active enzymes and genomes, respectively.

The *L. casei* FBL6 genome contained 119 genes in the CAZymes gene families as follows: 61 glycoside hydrolases (GHs), 40 glycosyltransferases (GTs), 3 carbohydrate esterases (CEs), 1 auxiliary activity (AA), 13 carbohydrate-binding modules (CBMs), and 1 polysaccharide lyase (PL) (Figure 4). The *L. chiayiensis* FBL7 genome contained 105 genes in CAZymes gene families: 60 GHs, 29 GTs, 3 CEs, 1 AA. 11 CBMs, and 1 PL. Among them, GH78, GT0, and CBM48 were only predicted in FBL7, not in other *L. chiayiensis* strains. The *L. zeae* FBL8 genome contained 115 genes in CAZymes gene families: 56 GHs, 43 GTs, 3 CFs, 1 AA, 9 CBMs, and 3 PLs. The results demonstrated that the carbohydrate utilization patterns of FBL6, FBL7, and FBL8 were different. GH127 (β-L-arabinofuranosidase) and GT111 (β-1,3-galactofuranosyltransferase) were only predicted in the FBL6 strain, whereas GH154 (β-glucuronidase) and GH43-11 (β-xylosidase) were only predicted in the FBL7 strain. Also, GH15 (glucoamylase), GH105 (unsaturated rhamnogalacturonyl hydrolase), and PL8 (hyaluronate lyase) were only predicted in the FBL8 strain. A previous study has reported that the larger the number of CAZymes encoding genes in the genome, the higher the probiotic potential for immune stimulation and pathogen defense (Zhang et al., 2018). FBL6, FBL7, and FBL8 exhibited a higher diversity of carbohydrate-activation enzymes, which were relatively abundant than other strains.

### Probiotic Properties Under *in vitro* Conditions

#### Acid and Bile Resistance

**FIGURE 5:**
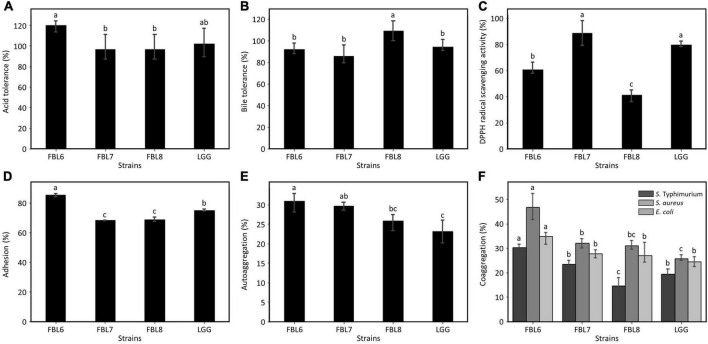
Evaluation of the probiotic properties of *L. casei* FBL6, *L. chiayiensis* FBL7, and *L. zeae* FBL8. **(A)** The acid and **(B)** bile tolerance of three strains. **(C)** The antioxidant activity and **(D)** adhesion activity of three strains. **(E)** The autoaggregation and **(F)** coaggregation of three strains on *S.* Typhimurium ATCC 14028, *S. aureus* ATCC 6538, and *E. coli* ATCC 43894. Data value represents the mean ± standard deviation (*n* = 3). Different letters on each bar indicate significant differences (*p* < 0.05).

The effects of probiotic strains depend on their ability to multiply in the gastrointestinal tract to exhibit functionality and survive in natural host defenses (Ye et al., 2020). For the cells to reach the intestine and exhibit functionality, probiotic strains must be resistant to low pH and bile salt (Yu et al., 2019). The ability of the strains to survive in simulated gastric juice and bile salt was evaluated *in vitro*. The survival of FBL6, FBL7, and FBL8 was not affected at all by the low-acid environment, and these strains exhibited survival rates of 96.7 ± 12.75% to 120.3 ± 6.14%, confirming the high stability against acid (Figure 5A). Also, the three strains showed survival rates of 85.4 ± 8.97% to 108.8 ± 9.10% at a high concentration of bile salt, indicating that they were not affected by bile salt to survive (Figure 5B). Therefore, these results suggest that three strains possess the characteristics of probiotics with high survival rates at low pH and bile concentration.

#### Antioxidant Activity

The DPPH radical scavenging activity of the strains was analyzed to observe the antioxidant effect, which is one of the probiotic properties. As a result, the three strains exhibited a DPPH scavenging potential (Figure 5C). There is no significant difference in DPPH radical scavenging activity between *L*. *chiayiensis* FBL7 strain and control strain, *L*. *rhamnosus* GG. The *L. casei* FBL6 and *L. zeae* FBL8 strains showed lower activities. The DPPH radical scavenging activities of the three strains ranged from 41.0 ± 4.62% to 88.6 ± 9.52%, and all strains exhibited antioxidant activity.

#### Antimicrobial Properties

The inhibitors produced by probiotic strains can be alternatives to antibiotics (Li et al., 2021). The antimicrobial properties of the three strains against the representative pathogenic strains were investigated. *L. casei* FBL6, *L. chiayiensis* FBL7, and *L. zeae* FBL8 had a broad antimicrobial spectrum (Table 3). These strains exhibited antimicrobial activity against Gram-negative bacteria and Gram-positive bacteria, such as *Listeria*, *B. cereus*, *E. coli*, and *Salmonella*. The antimicrobial activity of probiotic strains is mainly mediated by the production of some inhibitory substances, such as bacteriocins and lactic acid (Ye et al., 2020). These strains have genes involved in the production of antimicrobial peptides in the genomes, and it is presumed that these substances contributed to the inhibitory effect on pathogenic strains.

**TABLE 3:** Antimicrobial activity of *L. casei* FBL6, *L. chiayiensis* FBL7, and *L. zeae* FBL8.

Strains	Antimicrobial activity against pathogenic strains*^a^*					
	*L. monocytogenes* ATCC 19111	*B. cereus* ATCC 21772	*E. coli* O157:H7 ATCC 43894	*E. coli* O1:K1:H7 ATCC 11775	*S.* Enteritidis ATCC 4931	*L. ivanovii* ATCC 19119
*L. casei* FBL6	++	++	++	+++	+++	+++
*L. chiayiensis* FBL7	++	++	++	++	++	++
*L. zeae* FBL8	++	++	+++	+++	++	++
*L. rhamnosus* GG	+++	++	++	+++	+++	+++

*^a^The diameter of the inhibition zone was evaluated according to the following criteria: very strong (+++, ≥17 mm), strong (++, 11–17 mm), weak (+, 5–10 mm), no inhibition (–).*

#### Adhesion Ability

The adhesion ability of probiotic strain has been proven to vary depending on their species or strain because the production of proteinaceous secretory adhesion and the properties of bacterial cell membranes contribute to intestinal cell attachments (Yu et al., 2019). *L. casei* FBL6 exhibited an adhesion capability of 85.4 ± 1.37%, which is higher than that of the control strain, *L. rhamnosus* GG (Figure 5D). This result is consistent with a previous study demonstrating the probiotic *L. casei* strain’s high adhesion ability (Yu et al., 2019). *L. chiayiensis* FBL6 and *L. zeae* FBL7 strains demonstrated lower adhesion capabilities than *L. rhamnosus* GG but were similar to other probiotic strains, such as *L. plantarum* subsp. *plantarum* SW03, *L. rhamnosus* KCTC 12202BP, and *L. gasseri* 5R01 (Oh and Jung, 2015; Son et al., 2017; Oh et al., 2018). These results suggest that three strains have potentially fulfilled the adhesion capacity requirements for probiotics.

#### Autoaggregation and Coaggregation

Autoaggregation is the phenomenon of clumping of bacteria of the same strain and is correlated with the adhesion ability of the probiotic strain to the intestinal epithelial cells (Lee et al., 2014). This ability is a precondition for colonization and improved persistence in the gastrointestinal tract (Lee et al., 2014). The autoaggregation rates of FBL6, FBL7, and FBL8 were measured. The results indicated that all strains possessed autoaggregation phenotypes (Figure 5E) and showed higher autoaggregation than the control strain, namely *L. rhamnosus* GG, except for *L*. *zeae* FBL8. There is no significant difference in autoaggregation between *L*. *zeae* FBL8 and *L*. *rhamnosus* GG.

Coaggregation refers to clumping between two different species. The coaggregation ability of the probiotic and pathogenic strains may hinder colonization by pathogenic strains in the gut, allowing a host defense mechanism against infection (Lee et al., 2014). Coaggregation of FBL6, FBL7, and FBL8 strains with pathogenic strains was measured. All strains demonstrated coaggregation with three pathogenic strains compared with the control strain, *L. rhamnosus* GG, except for the coaggregation of FBL8 with *S*. Typhimurium. FBL6 showed the best coaggregation rate with *S. aureus* (46.8 ± 5.41%) (Figure 5F). The genomic analysis and *in vitro* studies suggest that these strains may have adhesion ability in human intestinal epithelial cells.

## Conclusion

This study was conducted to demonstrate the probiotic properties of *L. casei* FBL6, *L. chiayiensis* FBL7, and *L. zeae* FBL8 isolated from raw milk. The FBL7 and FBL8 strains are the first *L. chiayiensis* and *L. zeae* strains with phenotype and genotype studies. For the first time, this study revealed that *L. chiayiensis* and *L. zeae*, like *L. casei*, also exhibit probiotic properties. The genome sequencing and comparative genomic analysis data indicated that three strains play a role as a potential probiotic candidate with antibacterial activity and the ability to survive in environmental stresses, such as acid, bile, temperature, and oxidative stress. Comparative genomic analysis indicated the *L*. *casei* FBL6 carried a sakacin-P gene cluster that was not found in other *L*. *casei* genomes. Moreover, genes related to adaptation, such as carbohydrate-active enzymes and secondary metabolite gene clusters specific to the genomes of three strains, were also revealed. Our analysis results could provide a genetic basis for further investigation to show the three strains’ functional mechanism of probiotic characteristics. However, it is necessary to further elucidate its specific benefits for host health through *in vitro* and *in vivo* experiments.

## Data Availability Statement

The datasets presented in this study can be found in online repositories. The names of the repository/repositories and accession number(s) can be found below: NCBI AND CP074377.1, CP074378.1, CP074379.1.

## Author Contributions

EK and H-YK contributed to the conception and design of this study. EK and S-MY performed the genome analysis and comparative genomic analysis. S-MY and DK performed the *in vitro* probiotic test. EK prepared a draft manuscript. H-YK reviewed and edited the manuscript. All authors contributed to manuscript revision, read, and approved the submitted version.

## Conflict of Interest

The authors declare that the research was conducted in the absence of any commercial or financial relationships that could be construed as a potential conflict of interest.

## Publisher’s Note

All claims expressed in this article are solely those of the authors and do not necessarily represent those of their affiliated organizations, or those of the publisher, the editors and the reviewers. Any product that may be evaluated in this article, or claim that may be made by its manufacturer, is not guaranteed or endorsed by the publisher.

## Footnotes

1
https://www.cazy.org/
