# Supplementary material for: Complete Genome Sequencing and Comparative Genomics of Three Potential Probiotic Strains, Lacticaseibacillus casei FBL6, Lacticaseibacillus chiayiensis FBL7, and Lacticaseibacillus zeae FBL8
Source: Front Microbiol. 2022 Jan 7;12:794315. doi: 10.3389/fmicb.2021.794315 (PMC8777060; doi:10.3389/fmicb.2021.794315)
Supplement: Supplementary file 1 [file Data_Sheet_1.docx]

***Supplementary Materials***

**
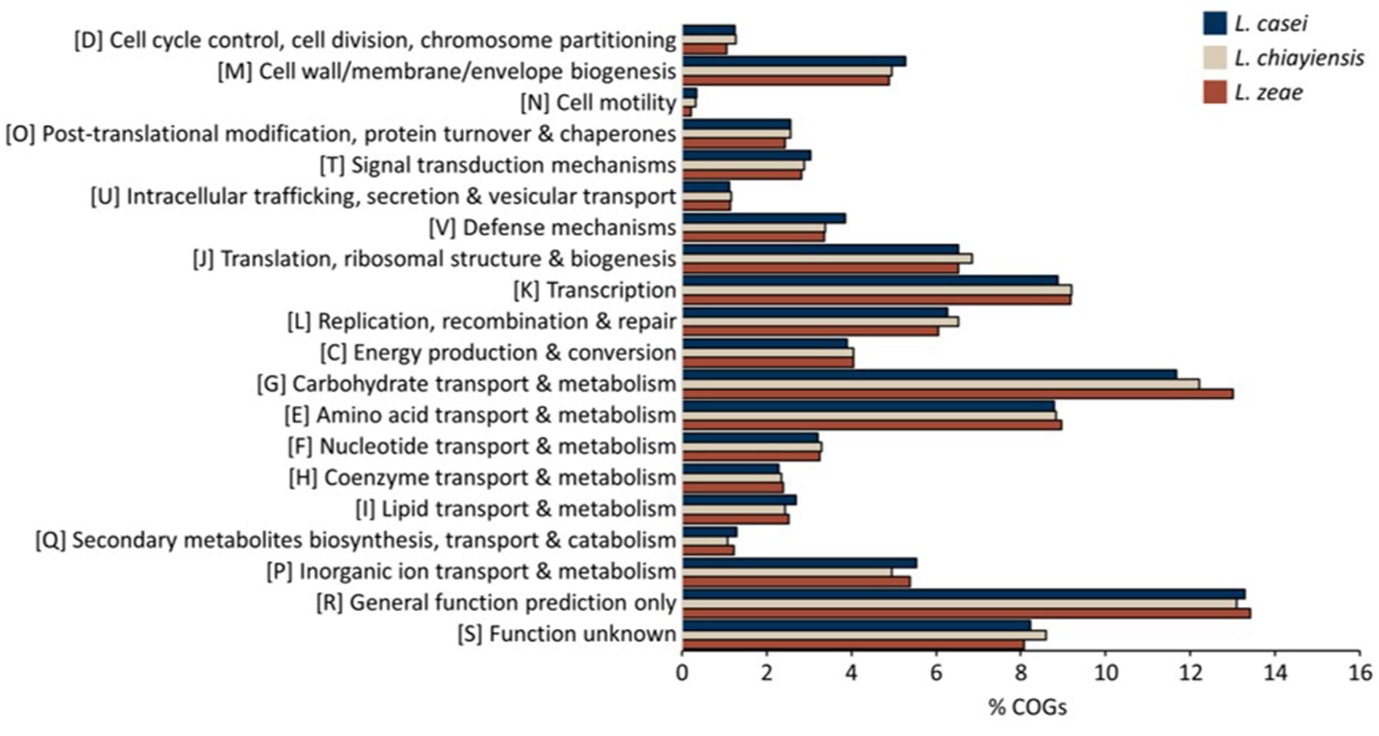
**

**Supplementary Fig. S1.** The number of genes assigned in COG categories. Blue, brown, and red bars represent COGs of the *L. casei* FBL6, *L. chiayiensis* FBL7, and *L. zeae* FBL8 genomes, respectively.


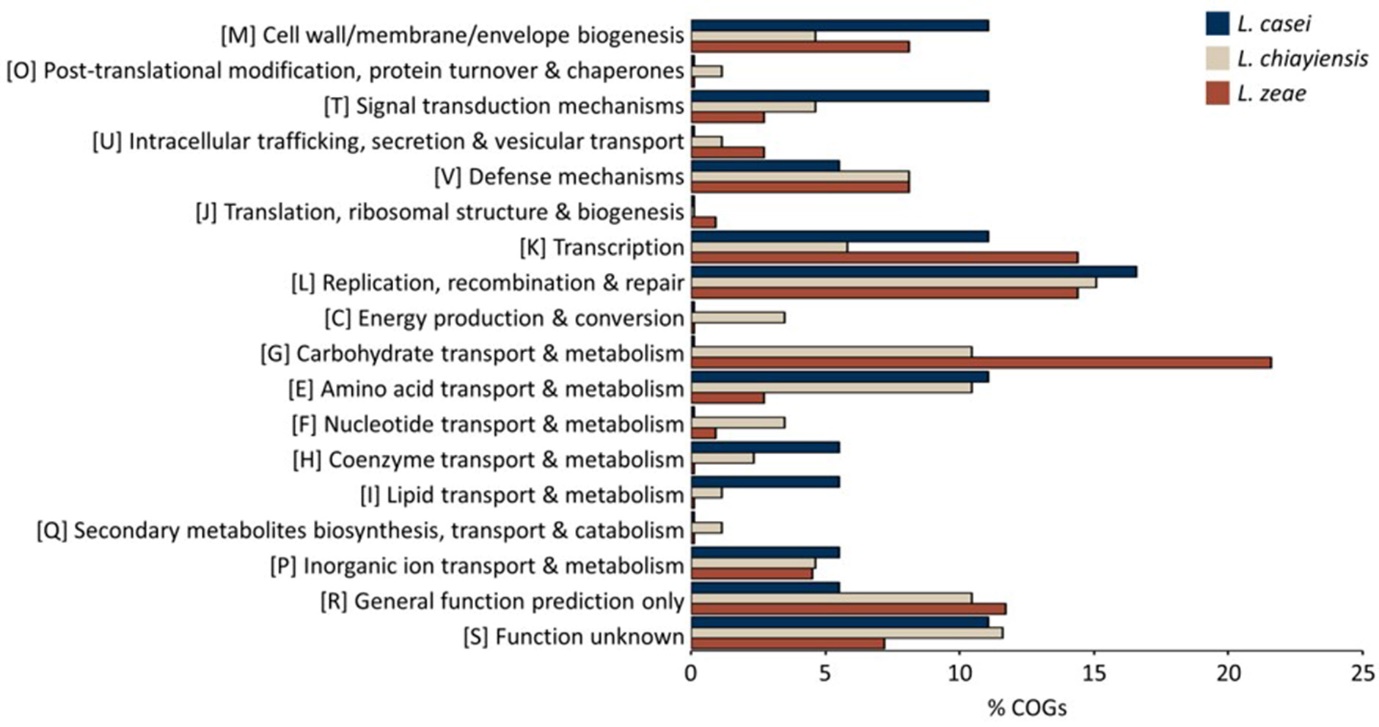
**Supplementary Fig. S2.** The number of unique genes in each strain assigned in COG functional categories. Black, gray, and white bars represent COGs of the *L. casei* FBL6, *L. chiayiensis* FBL7, and *L.* *zeae* FBL8 unique genes, respectively.


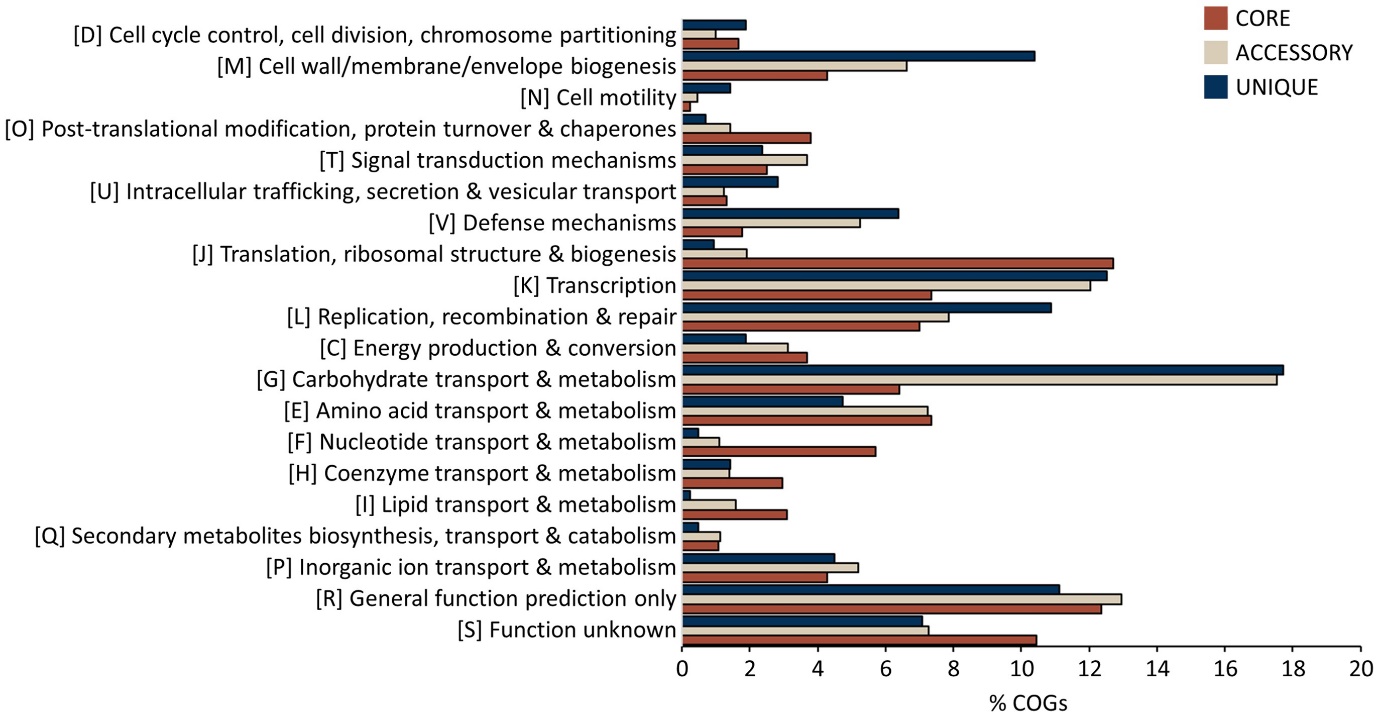


**Supplementary Fig. S3.** The number of genes assigned in COG categories for 86 *Lacticaseibacillus* strains. Red, brown, and blue bars represent COGs of core-genome, accessory-genome, and unique-genome, respectively.
